# Proton Pump Inhibitors Induced Hyponatremia in a Liver Transplanted Patient—The Role of Deprescribing: A Case Report and Literature Review

**DOI:** 10.3390/reports7020033

**Published:** 2024-05-06

**Authors:** Gianmarco Marcianò, Benedetto Caroleo, Luca Catarisano, Donatella Cocchis, Caterina Palleria, Giovambattista De Sarro, Luca Gallelli

**Affiliations:** 1 Operative Unit of Pharmacology and Pharmacovigilance, “Renato Dulbecco” University Hospital, 88100 Catanzaro, Italy; gianmarco.marciano3@gmail.com (G.M.); lucacatarisano@gmail.com (L.C.); palleria@unicz.it (C.P.); desarro@unicz.it (G.D.S.); 2 Operative Unit of Internal Medicine, Soverato Hospital, ASP7 Catanzaro, 88100 Catanzaro, Italy; benedettocaroleo@libero.it; 3 Department of Liver Transplants, Citta Della Salute Hospital, 10126 Torino, Italy; dcocchisd@alice.it; 4 Research Center FAS@UMG, Department of Health Science, University Magna Graecia, 88100 Catanzaro, Italy

**Keywords:** liver transplant, immunosuppressants, deprescribing, adverse drug reaction, hyponatremia, pantoprazole

## Abstract

Liver transplant patients are frail subjects due to lifelong therapy with immunosuppressants. In these patients, comorbidity and polytherapy increase the risk of adverse drug reactions. In this study, we report the development of hyponatremia, probably related to pantoprazole in a liver transplant patient. Sertraline dismission and treatment with sodium chloride did not improve clinical symptoms and laboratory levels. Pantoprazole dismission induced an improvement in clinical symptoms and the normalization of sodium levels. A five-month follow-up revealed the absence of clinical symptoms and normal serum sodium levels.

## 1. Introduction

Liver transplantation is a challenging clinical setting indicated in patients with a poor prognosis or a low quality of life. Hepatocarcinoma (HCC), end-stage liver disease (ESLD), and acute liver failure (ALF) are the most common indications [1]. After a transplant, immunosuppressants must be used to avoid tissue rejection, e.g., calcineurin inhibitors (tacrolimus, TAC; cyclosporine; CYS), mammalian target of rapamycin inhibitors (everolimus, and sirolimus), and inhibitors of purine and pyrimidine synthesis (mycophenolate mofetil; azathioprine) [2]. Alongside monoclonal antibodies, muronomab (that exerts its action against CD3), basiliximab, daclizumab (acting on the IL-2 receptor), anti-thymocyte globulin, and anti-lymphocyte globulin could also be used [2]. Even if these drugs show good clinical efficacy, their use can be related to the development of adverse drug reactions (ADRs) [3], including nephrotoxicity [2,4,5]. Proton pump inhibitors (PPI) are commonly prescribed to reduce gastric acidity in several clinical conditions (e.g., gastroesophageal reflux disease, peptic ulcer, Zollinger–Ellison syndrome, and Helicobacter pylori infections) [6,7]. However, their use is associated with several ADRs, i.e., gastrointestinal side effects, hematologic alterations, headaches, cutaneous reactions, fractures, nephritis, liver enzyme modifications, and electrolyte concentration changes [8,9]. In this study, we report a middle-aged liver-transplanted man developing hyponatremia during PPI treatment.

## 2. Detailed Case Description

**Figure 1:**
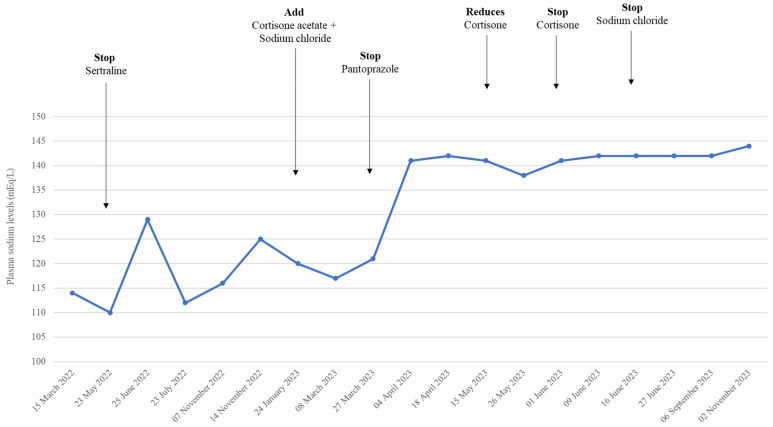
Time table of serum sodium levels in enrolled patient.

A 45-year-old liver-transplanted man came to our observation for clinical evaluation. The patient’s medical history showed that he had hypertension, subclinical hypothyroidism, and depression. The patient’s current treatment includes tacrolimus, mycophenolate, ramipril, acetylsalicylic acid, pantoprazole, furosemide, sertraline, and ursodeoxycholic acid. About 2 years ago, in December 2021, he received a cadaver liver transplantation and started treatment with tacrolimus. Three months later (15 March 2022), he experienced nausea, headache, asthenia, loss of appetite, and confusion, which led to his hospitalization. The laboratory results revealed hyponatremia (serum sodium levels of 114 mEq/L; the normal value is 135–140 mEq/L), with normal levels of plasma cortisol levels (56 ng/mL; the normal range is 50–250) and adrenocorticotropic hormone levels of 19 pg/mL (normal range 5–24); therefore, treatment with sodium chloride infusions was started (Figure 1).

Two months later (23 May 2022), the persistence of clinical signs and symptoms of hyponatremia, confirmed by laboratory tests (serum sodium levels of 110 mEq/L; urinary sodium of 181 mmol/24 h, normal range of 90–220; urinary cortisol of 247.3 mcg/24 h, normal range of 21–292), suggested stopping furosemide and sertraline without a change in sodium levels. Interestingly, antidiuretic hormone (ADH) levels of 11.3 pg/mL (0.5–7.6) were above the upper limit, even one week after sertraline deprescribing. Six months later (14 November 2022), a new follow-up confirmed the presence of hyponatremia (125 mEq/L) without other changes in laboratory findings or any other change in pharmacological treatment. About two months later (January 2023), due to the persistence of clinical symptoms, the patient came to our attention. A clinical evaluation excluded systemic diseases, while laboratory tests excluded the presence of organ rejection and confirmed the presence of hyponatremia (116 mEq/L). Psychiatric evaluation excluded psychogenic polydipsia, while endocrinologic consultancy suggested starting treatment with cortisone acetate (25 mg twice daily) and sodium chloride (500 mg twice daily). Two months later (27 March 2023), a new follow-up documented the presence of hyponatremia (117 Meq/L) (Figure 1). Consultants in clinical pharmacology suggested stopping pantoprazole (40 mg) and continuing sodium chloride infusions. One month later (18 April 2023), we documented a clinical and laboratory improvement (sodium levels of 141 mEq/L). Cortisone acetate use was discontinued after about 1 month, while sodium chloride use was stopped on 16 June 2023. A five-month follow-up (3 November 2023) documented normal levels of serum sodium (144 mEq/L) and the absence of clinical signs or symptoms of diseases. In this time interval, the levels of potassium (variation from 3.6 to 4.3 mEq/L), creatinine (from 0.50 to 0.70 mg/dL), and azotemia (from 15 to 32 mg/dL) were monitored. The Naranjo probability scale revealed a possible association between pantoprazole and hyponatremia (score 6).

## 3. Discussion

In this study, we report a liver-transplanted patient who developed severe hyponatremia during pantoprazole treatment. Severe hyponatremia (<120 mEq/L) is associated with gastrointestinal symptoms and anorexia, headache, and muscle cramps, as well as altered mental status, asthenia, agitation, seizures, and even comas [10]. Hyponatremia may also be associated with several complications, including falls, fractures, and myocardial infarction [9].

Several factors may determine the onset of hyponatremia (Table 1), including the use of medications like diuretics, antidepressants, anti-seizure drugs, antineoplastic agents, vasopressin analogues, antipsychotics, opioids, non-steroidal anti-inflammatory drugs (NSAIDs), cyclophosphamide, and proton pump inhibitors [10,11].

**Figure 2:**
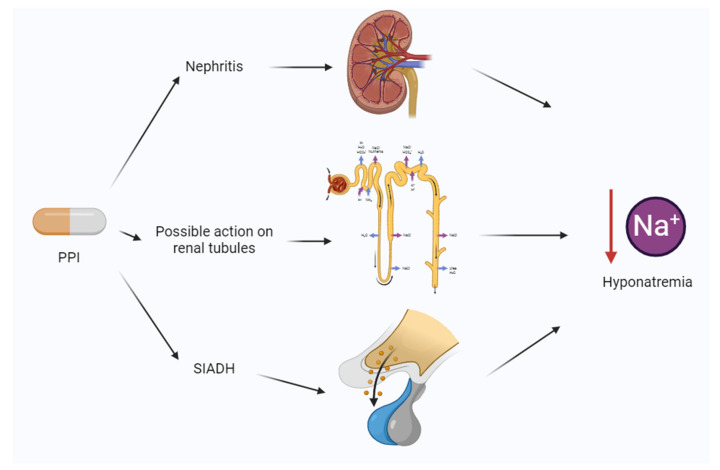
Mechanisms of protein pump inhibitor (PPI)-induced hyponatremia. SIADH, syndrome of inappropriate secretion of antidiuretic hormone.

In this context, the frequency of PPI-induced hyponatremia is often reported as low or undefined by labels [12,13]. Even if the mechanism of pantoprazole-induced hyponatremia is unclear [14], it could be generally related to SIADH, interstitial nephritis (more unlikely), or peripheral effects on renal tubules. This last mechanism, including the direct effect of renal tubular ion exchange, is unproven and mainly of a speculative nature (Figure 2).

Mennecier et al. [15] consider SIADH associated with PPIs a rare event and that its mechanism should be described in dedicated papers. Extensive sodium urinary excretion may be the main cause of hyponatremia. In general, the specific mechanism of PPIs-induced hyponatremia lacks evidence. Certainly, the presence of risk factors and of old age may favor this adverse event. Ganguli and colleagues [16] observed that the presence of age-related adrenal insufficiency; the overexpression of some cytokines, including interleukin-6 (that was associated with increased vasopressin secretion); or simply poor solute intake are relevant risk factors in the elderly. Nevertheless, frailty may be partially mediated in hyponatremia since experimental models of this condition led to sarcopenia.

Furthermore, diseases whose incidence increases with age, i.e., kidney disease and heart failure, are associated with body water decrease and hyponatremia. The consumption of drugs (antidepressants, diuretics) may exert an additive action with PPI. Other factors like vomiting, diarrhea, or drinking excessive quantities of water may be related to hyponatremia [17].

Naharcy et al. [18] reported an 80-year-old frail male patient admitted to the hospital with vertigo, nausea, and weakness. He had several comorbidities, including Parkinson’s disease, and managed reflux with pantoprazole for 3 years. Before starting pantoprazole, his natremia was 137 mEq/L, then progressively decreased to 129 mEq/L when he was hospitalized. After pantoprazole discontinuation, natremia gradually improved. It is important to remember that both elderly and Parkinson’s patients have a higher risk of SIADH.

Qureshi et al. [19] reported a case of a 56-year-old woman consuming omeprazole for four days for acid reflux. She presented to primary care for generalized weakness, which worsened in the following days with the addition of slurred speech. The analyses showed severe hyponatremia alongside encephalomyelitis (acute disseminated encephalomyelitis) in magnetic resonance imaging. Hypertonic saline, steroids, and omeprazole discontinuation improved the clinical status.

Buon et al. [20], in a retrospective study on 145 patients, highlighted a higher rate of hyponatremia in subjects consuming PPIs for at least 1 year in comparison to the rest of the population (31.3% vs. 9.3%). Since the authors did not describe a relationship between dose and hyponatremia, the use of tramadol was documented to be a risk factor.

Makunts et al. [21], analyzing the FDA Adverse Events Reporting System records on 42,537 patients treated with PPIs, revealed an increase in hyponatremia rate during the treatment with omeprazole, lansoprazole, and rabeprazole.

Falhammar and colleagues [14], evaluating the data of 14,359 patients with hyponatremia, showed an association with PPI use (lansoprazole excluded). Adjusted ORs (95% CI) for hospitalization due to hyponatremia, compared to controls, were as follows for recently initiated PPIs: esomeprazole 2.89 (2.21–3.79), omeprazole 2.67 (2.37–3.01), pantoprazole 2.06 (1.32–3.19), lansoprazole 1.19 (0.72–1.94), and any PPI 2.78 (2.48–3.11), with *p* < 0.05 for all PPIs lansoprazole and rabeprazole excluded. Only one individual had been newly initiated on rabeprazole and had been hospitalized due to hyponatremia. The results were not confirmed in a chronic setting in which the use of PPIs seems not to be related to hyponatremia (*p* > 0.05). It is legitimate to assume that the difference in the chemical structure of lansoprazole and the presence of compensation strategies by organisms in chronic settings may explain the absence of statistical significance. Lansoprazole may therefore be prescribed in patients with risk factors without forgetting interaction risks related to each PPI. However, this study had several limitations, including the design and the relatively small number of cases for some PPIs. Omeprazole and esomeprazole were the most represented drugs in the study, and therefore the results concerning these two compounds should be considered more robust.

More recently, Issa et al. [22] evaluated the association between hyponatremia and omeprazole or esomeprazole therapy in 11,213 subjects. The overall adjusted Odds Ratio was 1.23, whereas it was 6.87 in the first week and 1.10 in the course of ongoing treatment. Therefore, the authors concluded that the risk is mainly time-dependent, even if ongoing treatment is associated with a small increase in hyponatremia. A similar temporal relationship was described for other drugs (such as antiseizure drugs, antidepressants, thiazides, tramadol, and codeine). This may suppose a sort of feedback mechanism. This study had several limitations, including the possible underestimation of people using over-the-counter (OTC) drugs, the absence of complete information about sodium levels, and the possible insurgence of hyponatremia due to gastric ulcers or esophagitis.

Taken together, these data suggest a relationship between PPIs and hyponatremia at the beginning of the therapy. This may generate important clinical symptoms, as shown in our case. Another point is related to the etiology of hyponatremia in patients consuming PPI. Several authors suggest that SIADH is the most probable option [14], and it seems to be the most probable option in our case, according to the persistence of increased ADH levels one week after the cessation of sertraline. It is important to underline that our patient used tacrolimus to prevent liver rejection, and this could have increased the risk of hyponatremia. However, tacrolimus plasma levels were in the normal range. Similarly, all the drugs possibly implied in the determination of the clinical signs and symptoms were deprescribed according to the patient’s condition. Holding into account other studies, we may also suppose an alternative mechanism: the action on renal tubules may be mediated through the inhibition of transporters or cotransporters. The action of PPIs on the SLC22A family, in particular on organic cation transporter (OCT) proteins, has been a matter of discussion in the literature [23]. Nevertheless, little evidence concerning their action on inorganic cation transport has been described. Several proteins are involved in Na+ reabsorption in renal tubules, and PPIs may eventually inhibit a specific but unknown target [24]. In the present study, the urinary sodium level was in the normal range. Therefore, the normal level of sodium in the urine and its reduction in blood suggest a third actor, probably the renal interstitium [25]. However, no evidence of kidney injury was proven. Despite all these speculations, SIADH appeared to be the most probable mechanism in our case.

Another interesting argument in our case is that hyponatremia induced by PPI lasted for a long time interval (more than a year), differently from the literature evidence.

Altogether, this case report shows a clear causal association between PPI therapy and hyponatremia. The clinical severity of this condition may vary greatly depending on the subjects and range of symptoms. Physicians must aim to restore sodium levels and manage or remove causal factors. New prospective studies are required to describe the exact incidence of this side effect, differentiating the single risk associated with each specific PPI. Moreover, treatment duration and the possible role of drug–drug interaction must also be analyzed.

## Figures and Tables

**Table 1:** Causes of hyponatremia[10].

Hypovolemic Hyponatremia	Hypervolemic Hyponatremia	Euvolemic Hyponatremia
Diarrhea or vomiting Diuretics Third spacing of fluids (pancreatitis, hypoalbuminemia, small bowel obstruction) Mineralocorticoid deficiency Nephropathies Cerebral salt-wasting syndrome (urinary salt wasting, possibly caused by increased brain natriuretic peptide)	Extrarenal causes (congestive heart failure, cirrhosis) Iatrogenic Renal causes	Addison’s disease Drugs High fluid intake in conditions like primary polydipsia or potomania Hypothyroidism Medical testing related to excessive fluids, such as a colonoscopy or cardiac catheterization. Syndrome of inappropriate antidiuretic hormone (SIADH)

## Data Availability

The original contributions presented in the study are included in the article, further inquiries can be directed to the corresponding author.
